# An objective structural and functional reference standard in glaucoma

**DOI:** 10.1038/s41598-021-80993-3

**Published:** 2021-01-18

**Authors:** Eduardo B. Mariottoni, Alessandro A. Jammal, Samuel I. Berchuck, Leonardo S. Shigueoka, Ivan M. Tavares, Felipe A. Medeiros

**Affiliations:** 1 Vision, Imaging and Performance (VIP) Laboratory, Department of Ophthalmology, Duke Eye Center, Duke University, 2351 Erwin Rd, Durham, NC 27701 USA; 2 Department of Ophthalmology, Federal University of São Paulo, São Paulo, Brazil; 3 Department of Statistical Science and Forge, Duke University, Durham, NC USA; 4 Department of Electrical and Computer Engineering, Pratt School of Engineering, Duke University, Durham, USA

**Keywords:** Glaucoma, Diagnosis, Medical imaging

## Abstract

The current lack of consensus for diagnosing glaucoma makes it difficult to develop diagnostic tests derived from deep learning (DL) algorithms. In the present study, we propose an objective definition of glaucomatous optic neuropathy (GON) using clearly defined parameters from optical coherence tomography and standard automated perimetry. We then use the proposed objective definition as reference standard to develop a DL algorithm to detect GON on fundus photos. A DL algorithm was trained to detect GON on fundus photos, using the proposed objective definition as reference standard. The performance was evaluated on an independent test sample with sensitivity, specificity, area under the receiver operating characteristic curve (AUC), and likelihood ratios (LR). The test sample had 2118 fundus photos from 585 eyes of 405 individuals. The AUC to discriminate between GON and normal was 0.92 with sensitivity of 77% at 95% specificity. LRs indicated that the DL algorithm provided large changes in the post-test probability of disease for the majority of eyes. A DL algorithm to evaluate fundus photos had high performance to discriminate GON from normal. The newly proposed objective definition of GON used as reference standard may increase the comparability of diagnostic studies of glaucoma across devices and populations.

## Introduction

Despite recent advances in functional and structural assessment of glaucoma, its diagnosis remains a challenging task. Most individuals with the disease remain undiagnosed, while many healthy subjects are misdiagnosed and receive unnecessary treatment.

The lack of consensus on a reference standard for diagnosing glaucoma makes it difficult to evaluate newly proposed diagnostic tools. In recent years, advances in artificial intelligence (AI) have opened up the possibility for automated diagnoses of eye diseases using fundus photography. Deep learning (DL) algorithms have proven successful in detecting signs of diabetic retinopathy in fundus photos, achieving high accuracy when compared to a reference standard of human graders^1^. However, while the diagnosis of diabetic retinopathy is generally unequivocal, the same cannot be said for assessing the presence of glaucomatous damage, even for well-experienced specialists^2–4^. Notably in the early stages of the disease, it can be very difficult, if not impossible, to ascertain the presence of glaucoma on cross-sectional assessment of fundus photos. This is largely due to the wide variation in the appearance of optic discs, as well as to the lack of a precise definition of what the “characteristic” features of glaucoma are. This lack of a clear definition of structural glaucoma questions the validity and usefulness of attempts to develop DL models to replicate human gradings for glaucoma diagnosis from fundus photos.

Spectral-domain optical coherence tomography (SDOCT) provides objective and reproducible quantitative assessment of neural loss in glaucoma. The finding of correspondence between SDOCT and visual field abnormalities detected by standard automated perimetry (SAP) greatly enhances the specificity for diagnosing glaucomatous damage. Although subjective assessment of correspondence between structural and functional loss has been proposed as reference standard in diagnosing glaucoma damage^5^, to date, no objective definition of glaucomatous optic neuropathy (GON) has been proposed that incorporates both SDOCT and SAP and that could be applied for evaluation of artificial intelligence diagnostic algorithms.

In the present study, we propose an objective definition of GON using clearly defined structural and functional parameters from SDOCT and SAP. We then develop and validate a DL algorithm to detect GON on fundus photos, using the proposed objective definition as the reference standard. As such, to the best of our knowledge, the present work represents the first attempt to develop and validate an AI model to diagnose structurally and functionally defined GON using fundus photos.

## Results

Initially, SDOCT and SAP tests acquired within 180 days were paired and classified as having glaucoma or normal according to the objective reference standard (Table 1), described in detail in “Methods”. Pairs that did not meet criteria for either GON or normal were considered as suspects and were not included in the development of the DL algorithm. Then, the objective reference standard was assigned as label to the closest fundus photos acquired within 180 days of the SDOCT.

**Table 1:** Summary of criteria for the objective definition of glaucomatous optic neuropathy (GON).

Glaucomatous optic neuropathy (GON)		
	SDOCT	SAP
Global loss	Global RNFL thickness outside normal limits	GHT outside normal limits or PSD P < 5%
Localized loss	RNFL thickness outside normal limits in at least one superior sector (temporal superior and/or nasal superior)	Inferior MD P < 5%
	RNFL thickness outside normal limits in at least one inferior sector (temporal inferior and/or nasal inferior)	Superior MD P < 5%
**Normal**		
Normal	RNFL thickness within normal limits for all sectors and global	PSD probability not significant (P > 5%) and GHT within normal limits

Structural and functional criteria are derived from spectral-domain optical coherence tomography (SDOCT) and standard automated perimetry (SAP) results, respectively. To be considered glaucoma, it was necessary to meet the criteria for global or localized loss. To be considered normal, it was required that both SDOCT and SAP results were normal. SDOCT-SAP pairs that did not meet the criteria for either groups were considered suspects.

*SDOCT* spectral-domain optical coherence tomography, *SAP *standard automated perimetry, *RNFL* retinal nerve fibre layer, *GHT *glaucoma hemifield test, *PSD *pattern standard deviation, *MD *mean deviation.

The dataset comprised of 9830 fundus photos from 2927 eyes of 2025 individuals and was randomly split (at the individual level) into training/validation (80%) and test (20%) samples. The training/validation sample had 7712 fundus photos from 2342 eyes of 1620 subjects; the test sample had 2118 fundus photos from 585 eyes of 405 subjects. Of the 585 eyes in the test sample, 305 (52%) had GON and 280 (48%) were normal according to the objective reference standard. The median SAP mean deviation (MD) was − 7.5 and 0.2 dB for the glaucoma and normal groups, respectively; while mean global retinal nerve fibre layer (RNFL) thickness was 67.0 and 98.3 µm, respectively. Table 2 shows demographic and clinical characteristics for the eyes in the study.

**Table 2:** Demographic and clinical information of eyes and individuals included in the study.

	Training/validation		Test	
	Glaucoma	Normal	Glaucoma	Normal
Optic disc photos, no.	3980	3732	1061	1057
Eyes, no.	1254	1088	305	280
Subjects, no.	876	744	213	192
Age, mean (SD), years	61.7 (15.5)	53.2 (15.6)	62.8 (15.8)	52.9 (15.7)
Female, no. (%)	471 (53.7)	482 (64.8)	104 (48.8)	135 (70.3)
**Race, no. (%)**				
African American	274 (31.3)	228 (30.6)	65 (30.5)	55 (28.6)
Caucasian	497 (56.7)	462 (62.1)	121 (56.8)	122 (63.5)
Others	105 (12.0)	54 (7.3)	27 (12.7)	15 (7.8)
SAP MD, median (IQR), Db	− 6.58 (− 15.27, − 3.14)	0.06 (− 0.75, 0.84)	− 7.50 (− 11.96, − 3.67)	0.24 (− 0.55, 0.96)
Superior SAP MD, median (IQR), dB	− 6.48 (− 15.89, − 2.89)	0.12 (− 0.79, 1.00)	− 6.54 (− 14.85, − 3.04)	0.23 (− 0.58, 1.12)
Inferior SAP MD, median (IQR), dB	− 5.64 (− 14.73, − 2.79)	0.08 (− 0.81, 0.83)	− 6.12 (− 12.42, − 2.77)	0.15 (− 0.54, 0.84)
SAP PSD, mean (SD), dB	6.5 (4.0)	1.4 (0.2)	6.8 (3.9)	1.4 (0.2)
RNFL global thickness, mean (SD), µm	66.3 (16.4)	98.5 (10.3)	67.0 (15.9)	98.3 (11.5)
DL probability, median (IQR), %			99.8 (87.5, 100.0)	0.03 (0.0, 2.6)

*No.*  number, *SD* standard deviation, *SAP *standard automated perimetry, *MD* mean deviation, *IQR* interquartile range, *PSD* pattern standard deviation, *RNFL* retinal nerve fibre layer, *DL* deep learning.

**Figure 1:**
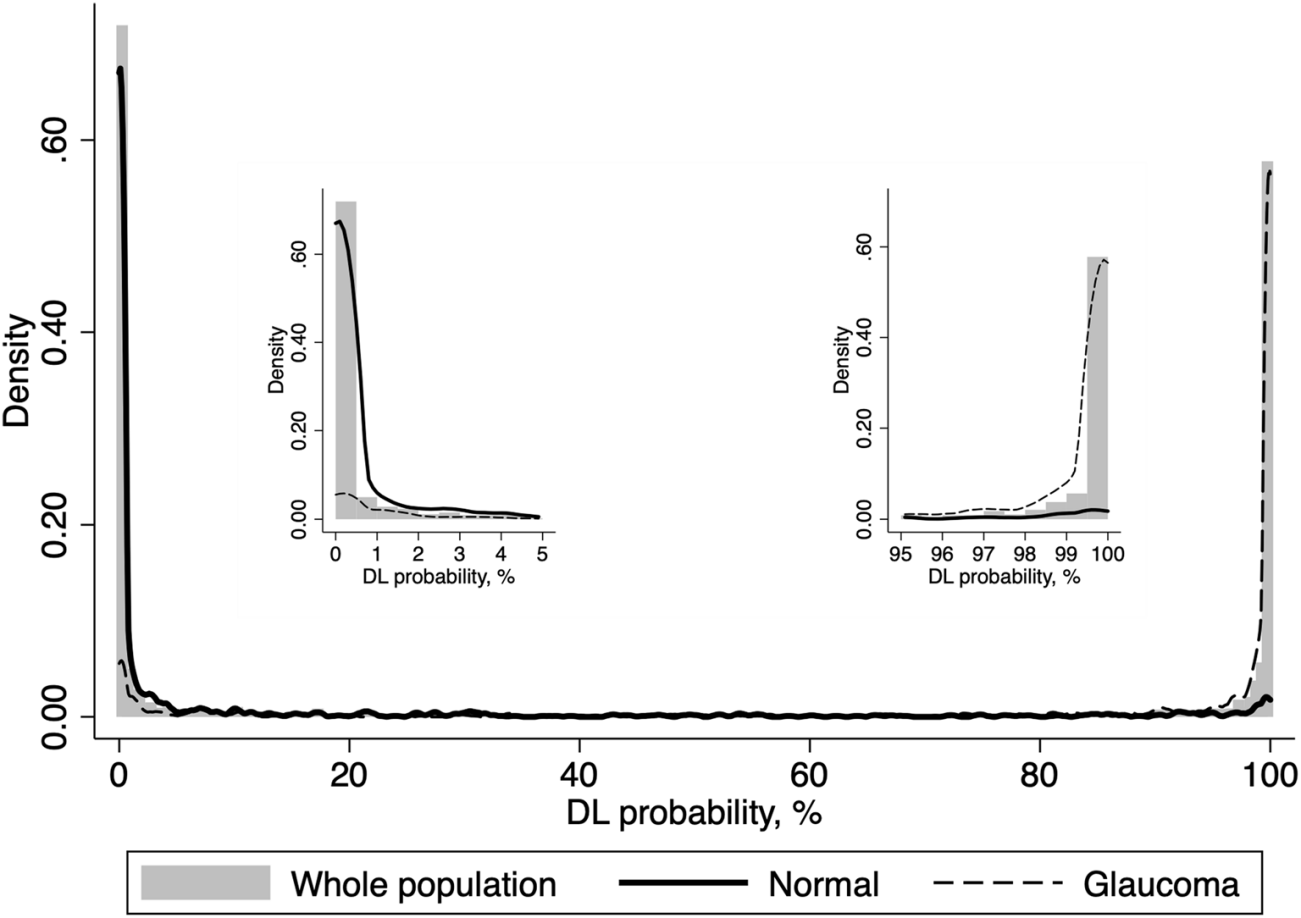
Distribution of the deep learning (DL) probabilities of glaucoma for the photos included in the test sample. The overall distribution is represented by shaded bars. Tails of the distribution (left, low probability of glaucoma; and right, high probability of glaucoma by the DL algorithm) are shown in detail. Photos classified as normal by the objective criteria proposed in the study had DL probabilities of glaucoma concentrated closer to zero (solid line). For photos classified as glaucoma, the DL probabilities were concentrated closer to one hundred (dotted line). *DL* deep learning.

A DL algorithm was trained to evaluate fundus photos and predict the classification GON versus normal, assigned according to the objective classification of the SDOCT-SAP pair. After the training process, the DL algorithm was used to yield the probability of GON of each fundus photo in the test sample. The median DL probability of glaucoma (interquartile range [IQR]) assigned to fundus photos with GON and normal were 99.8% (87.5, 100.0%) and 0.03% (0.0, 2.6%), respectively (P < 0.001). Figure 1 illustrates the distribution of DL probabilities.

**Figure 2:**
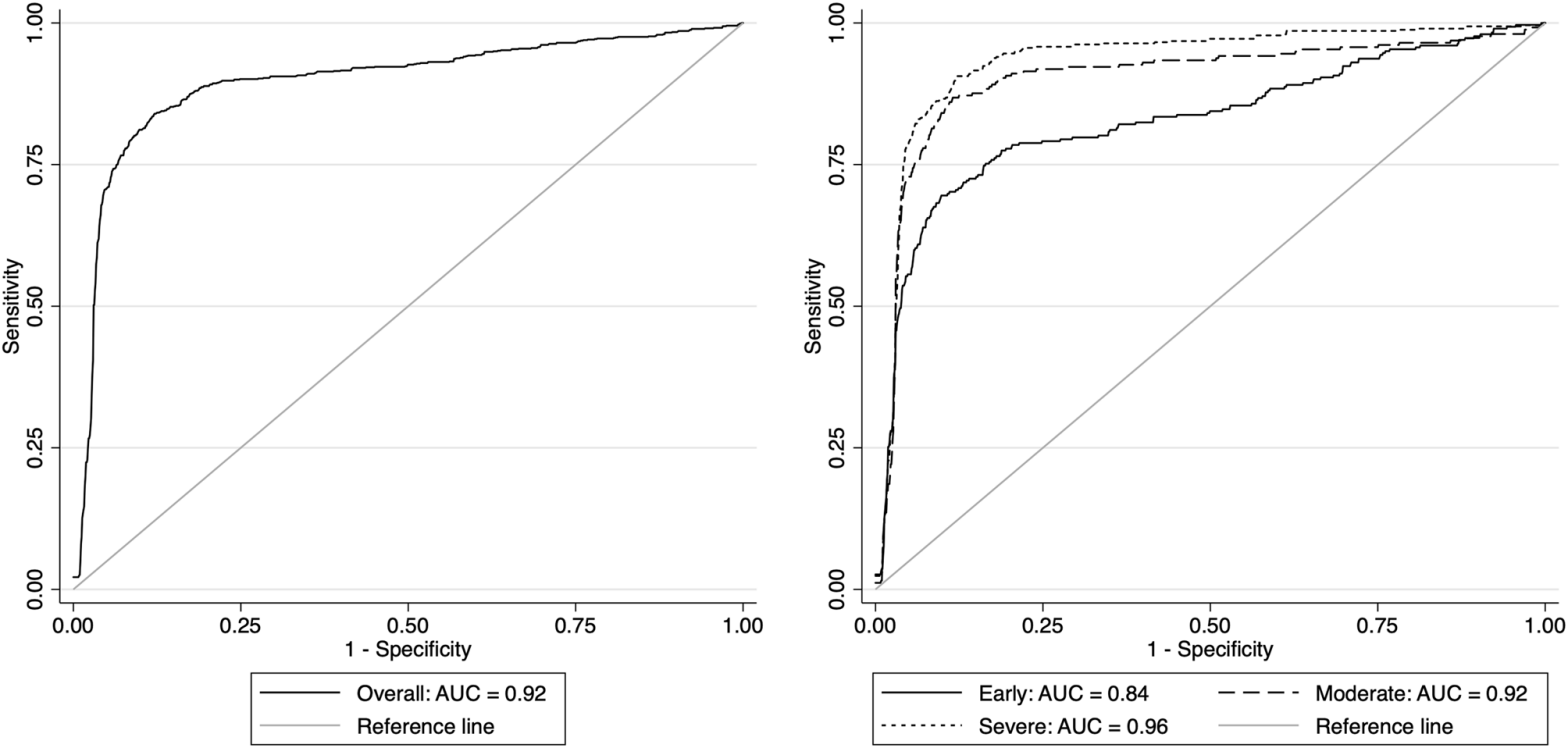
Age-adjusted receiver operating characteristic (ROC) curves illustrating the ability of the algorithm to discriminate between normal and glaucoma for all photos (overall) and at different levels of disease severity, according to the Hodapp–Parrish–Anderson classification of severity, based on visual field damage. *AUC* area under the ROC curve.

**Figure 3:**
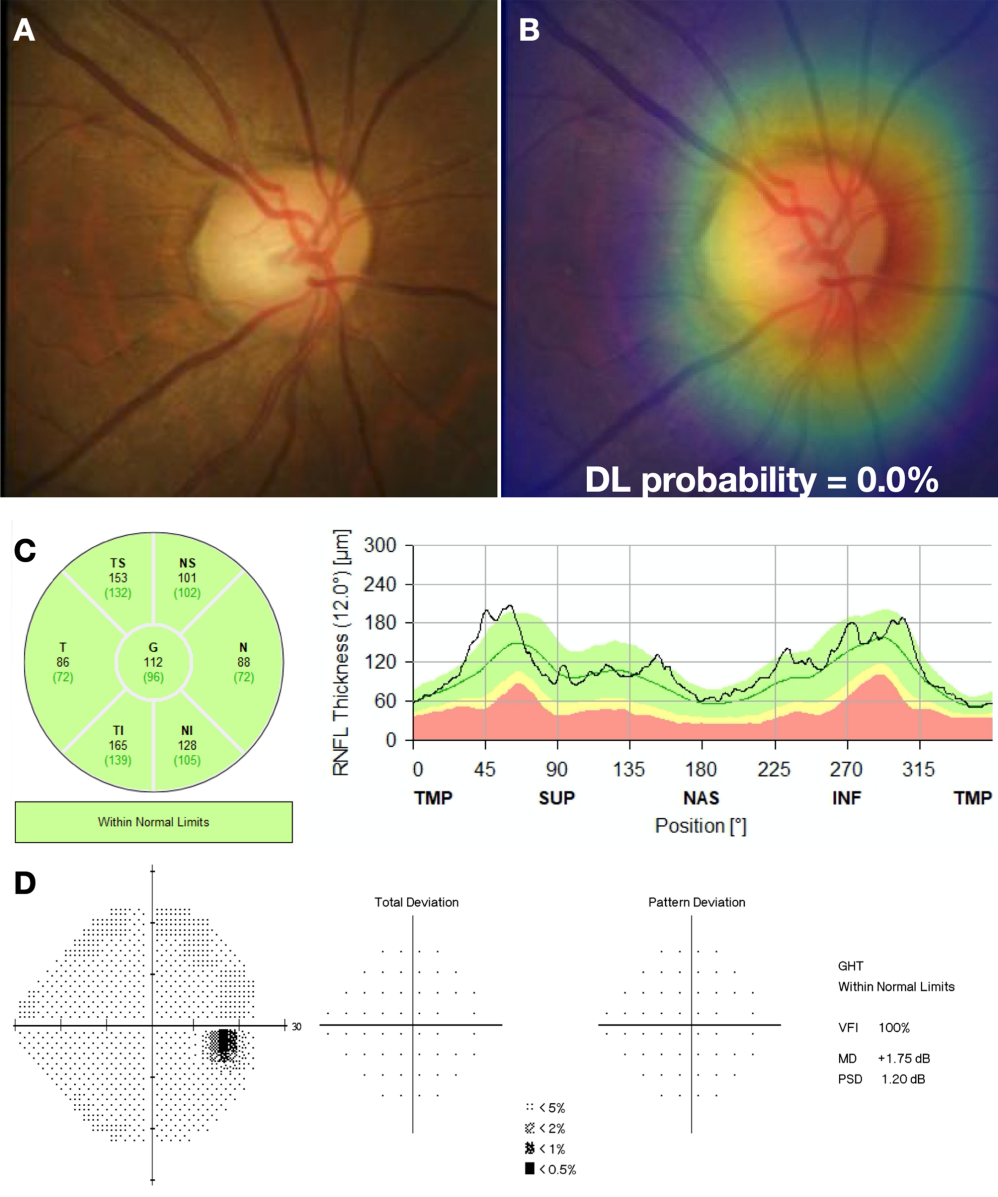
Example of a normal case included in the study. **(A)** illustrates the fundus photo used as input for the deep learning (DL) algorithm, which predicted a glaucoma probability of 0.0%. **(B)** shows the regions that were most important for the classification, in which the heatmap diffusely highlights the optic disc. **(C)** illustrates the spectral domain optical coherence tomography and **(D)** the standard automated perimetry, both without abnormalities.

**Figure 4:**
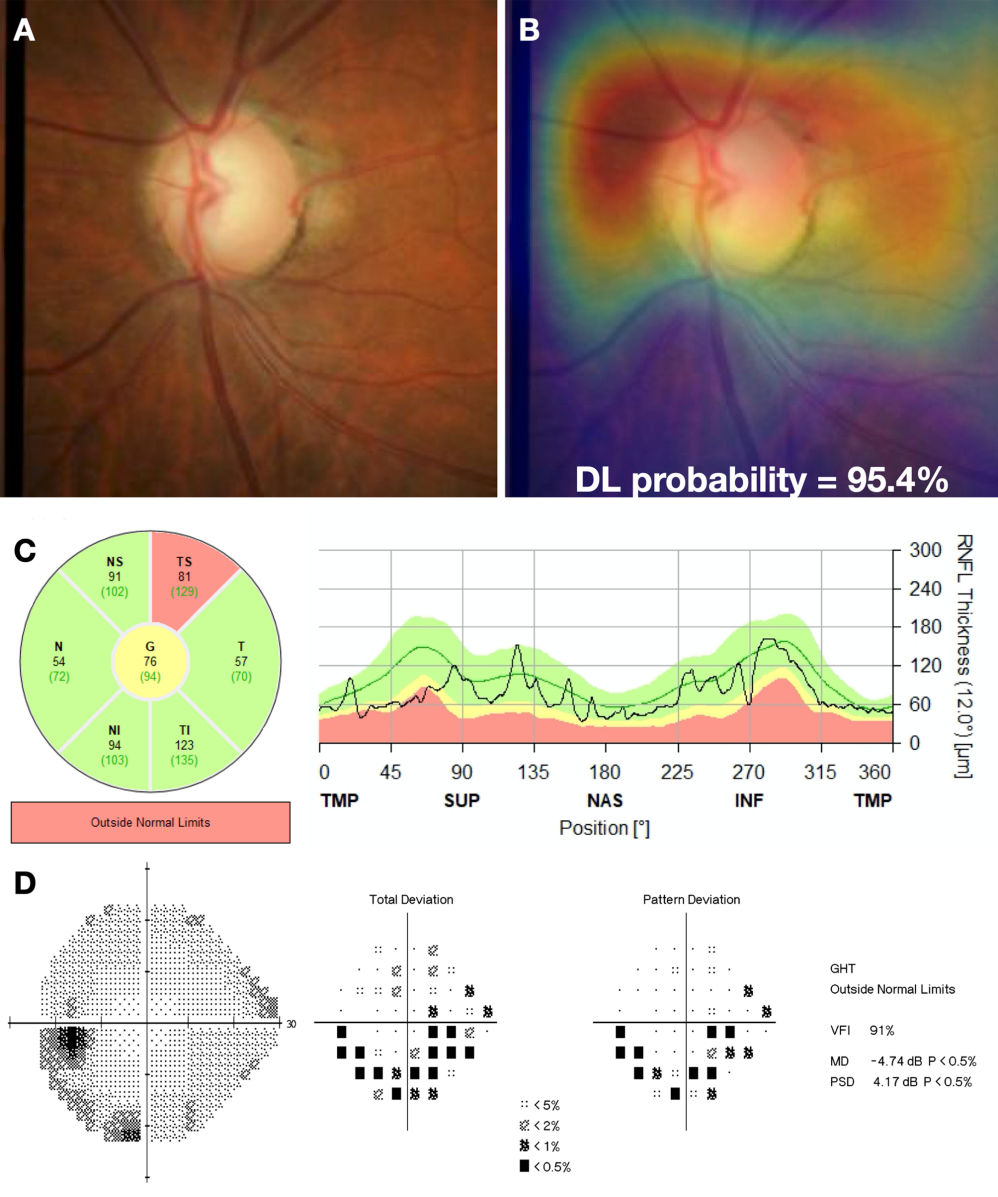
Example of a glaucoma case included in the study. **(A)** illustrates the fundus photo used as input for the deep learning (DL) algorithm, which predicted a glaucoma probability of 95.4%. **(B)** shows the regions that were most important for the classification, in which the heatmap highlights the superior half of the optic disc and peripapillary region. **(C)** illustrates the spectral domain optical coherence tomography with a temporal superior retinal nerve fibre layer defect, with a corresponding inferior arcuate defect in the standard automated perimetry **(D)**.

We investigated the performance of the DL algorithm to discriminate between fundus photos with GON and normal using receiver operating characteristic (ROC) curve and the area under the ROC curve (AUC) as well as sensitivity and specificity were used to summarize the diagnostic ability. The DL algorithm had an age-adjusted AUC of 0.92 (95% confidence interval [CI]: 0.88, 0.95) in the test sample. For a 95% specificity, the model had 77.3% sensitivity. The AUC values increased with worse levels of disease severity, achieving maximal age-adjusted AUC of 0.96 and sensitivity of 85.1% (at 95% specificity) among eyes with severe glaucoma. In Fig. 2 and Table 3, ROC curves and AUC values, as well as sensitivities at 95% specificity, are presented across levels of disease severity. Figures 3 and 4 illustrate eyes included in the study, classified as normal and as glaucomatous, respectively.

**Table 3:** Area under the receiver operating characteristic curve (AUC) and sensitivity at 95% specificity for different levels of disease severity, according to the Hodapp–Parrish–Anderson classification of glaucoma severity, based on visual field damage.

	Number of tests (%)	AUC (95% CI)	Sensitivity at 95% specificity
Overall	2,118 (100)	0.92 (0.88, 0.97)	77.3%
Normal	1,057 (49.9)		
Glaucoma			
Early	302 (14.3)	0.84 (0.77, 0.91)	60.4%
Moderate	258 (12.2)	0.92 (0.88, 0.97)	81.8%
Severe	501 (23.7)	0.96 (0.93, 0.98)	85.1%

*AUC* area under the receiver operating characteristic curve, *CI* confidence interval.

Likelihood ratios (LR) were calculated to assess the impact of the results of the DL algorithm in changing the probability of disease^6^. Table 4 presents the LRs for different intervals of DL probability of GON, identifying meaningful intervals for the risk of the disease. Test results with DL probability > 99% or > 99.9% were associated with large changes in increasing the probability of disease (LRs of 10.4 and 38.2, respectively); whereas test results with DL probability < 0.1% were associated with large change in decreasing the probability of disease (LR of 0.06). Any of these test results would provide strong evidence regarding the presence or absence of GON. As Table 4 shows, other test results would also increase or decrease the probability of disease, but provide less conclusive evidence, which could still be helpful depending on the setting of application of the test.

**Table 4:** Likelihood ratios for different intervals of Deep Learning probabilities of glaucoma.

DL probability, %	Glaucoma, no. (%)	Normal, no. (%)	Total, no. (%)	Likelihood ratio
0–0.1	38 (3.6)	604 (57.1)	642 (30.3)	0.06
0.1–1	32 (3.0)	141 (13.3)	173 (8.2)	0.23
1–10	57 (5.4)	116 (11.0)	173 (8.2)	0.49
10–50	70 (6.6)	86 (8.1)	156 (7.4)	0.81
50–90	78 (7.4)	47 (4.4)	125 (5.9)	1.65
90–99	141 (13.3)	36 (3.4)	177 (8.4)	3.90
99–99.9	146 (13.8)	14 (1.3)	160 (7.6)	10.4
99.9–100	499 (47.0)	13 (1.2)	512 (24.2)	38.2

*DL *deep learning, *No.*  number.

A total of 4899 fundus photos from 1648 eyes of 1085 individuals did not meet criteria for GON or normal and were, therefore, classified as suspects. When applied to this group, the DL algorithm predicted a median probability of GON (IQR) of 6.4% (0.2, 82.4%). These eyes had median (IQR) SAP MD of − 1.82 dB (− 4.32, − 0.31) and mean (SD) global RNFL thickness of 94.4 µm (15.7). When suspect eyes were divided in quartiles according to the DL probability, those in the highest quartile (higher probability) had significantly lower global RNFL thickness measurements than those in the lowest quartile (90.3 [16.8] vs. 98.7 µm [14.2]; P = 0.019] and also worse SAP MD, although the difference was not statistically significant (− 2.3 [− 5.4, − 0.7] vs. − 1.2 dB [− 3.3, 0.2]; P = 0.390). Supplementary Figures S1 and S2 illustrate eyes that were classified as suspects.

In order to assess the validity of the objective classification, a sample of 50% of individuals from the test sample (203) was randomly selected to be subjectively evaluated by glaucoma specialists, which included 725 SDOCT-SAP pairs from 357 eyes. The overall weighted agreement between the objective reference standard and the subjective classification was 95.2%, with a weighted kappa of 0.87 (Supplementary Table S1), indicating excellent agreement. Of note, the previously trained DL model had age-adjusted AUC = 0.92 to differentiate photos subjectively classified as normal versus glaucoma, with a sensitivity of 74.9% at 95% specificity (Supplementary Table S2).

## Discussion

In the present study, we developed novel objective criteria for defining GON, based on the correspondence of structural and functional measures. We then used the objective criteria as reference standard to train a successful DL algorithm to estimate the probability of GON from optic disc photos.

Fundus photos are an inexpensive and portable method to image the optic disc. Automated AI algorithms have been developed to analyse fundus photos for glaucoma diagnosis. However, most previous works have relied on human gradings to establish the reference standard, an approach that presents several limitations. Human gradings tend to over- and underestimate the likelihood of glaucoma on fundus photos^7^, and, even among specialists, there is poor reproducibility and low interobserver agreement^2–4^. Labelling a large number of fundus photos to be used as reference is cumbersome and time consuming, with the criteria used for labelling varying tremendously across studies.

In order to avoid subjective biases and low accuracy from human labelling, we proposed the use of an objective definition of GON, based on quantitative metrics of SAP and SDOCT. The DL algorithm trained to predict such classification from fundus photos showed high accuracy, with AUC of the DL of 0.92, improving to 0.96 for eyes classified as having severe disease. This performance makes the DL algorithm particularly useful for screening of moderate and severe glaucoma, which require prompt referral, but also with a high specificity, so as not to overwhelm the healthcare system with false-positive referrals. One could argue that the algorithm still misses about 20% of cases of glaucoma. However, given that previous population-based studies show that over 90% of patients with glaucoma remain undiagnosed in developing areas^8,9^, such a simple and inexpensive approach could potentially help reduce the burden of disability from glaucoma.

In contrast to sensitivity and specificity, which are difficult to apply in practice, LRs can be readily incorporated into clinical decision-making and better illustrate the potential of the DL algorithm for impacting diagnosis. In our study, DL probability values lower than 0.1% or greater than 99% were associated with large changes in the post-test probability, providing major conclusive changes regarding presence or absence of damage from glaucoma. To illustrate, consider the application of the test in a situation of screening, where the overall prevalence of glaucoma would be estimated at 5%^8,9^. This would then be considered the pre-test probability. If this patient is tested and gets a result with DL probability greater than 99.9% (LR of 38.2), this would bring the post-test probability to 66%, indicative of the need for referral.

LRs help to understand the usefulness of the test under different circumstances as well. For example, when applied to the evaluation of suspects in clinical practice or during opportunistic screening, levels of pre-test probability of disease would often be significantly higher, as those patients would most likely have clinical findings raising the probability of disease, such as family history or suspicious optic disc appearance. For example, if a patient has a pre-test probability of 50%, a test result with DL probability of 0.1% (LR = 0.06) would bring the post-test probability to just 5.7%. On the other hand, if a test result with DL probability of 99% were obtained, the post-test probability would jump to 91%. Importantly, DL probabilities below 0.1% or higher than 99% occurred in 62% of the overall sample, indicating that the test would provide conclusive evidence in a majority of patients.

The confidence of the DL predictions from fundus photos in classifying glaucoma damage, provides support for the objective reference standard used in our study. Conceivably, a weak reference standard would not elicit such strong agreement with predictions of glaucoma from fundus photos. We also further evaluated the objective reference standard by assessing its agreement with subjective gradings by glaucoma specialists, observing an overall weighted kappa of 0.87, indicative of excellent agreement. Interestingly, when the DL model trained against the objective standard was used to predict the subjective gradings, the AUC was identical at 0.92. This result is indicative of the potential for the objective parameters used in our study to replicate final decisions made by clinicians when interpreting SDOCT and SAP for diagnosis.

It is important to note that only eyes labelled as healthy or glaucoma by the objective definition were included in the development of the DL model. This was done to avoid including suspect eyes that had uncertain diagnosis and, therefore, estimates of diagnostic accuracy would be more relevant in a screening context. In fact, focusing on detecting very early stages of disease is unnecessary from a screening point of view. From a public health standpoint, detecting a patient before the symptomatic presentation is considered an early diagnosis. As most patients with glaucoma only present with symptoms at an advanced stage of the disease, almost any stage of glaucoma is considered an early diagnosis from a screening point of view. Glaucoma has relatively low prevalence in the general population and it can be challenging to discriminate between very early disease and normal variation. Therefore, attempting to identify very early cases on screening programs will likely lead to failure. Focusing on detecting cases that are well-established, while still possibly asymptomatic, will lead to much improved diagnostic accuracy and effectiveness. Of note, when applied to evaluate suspect cases, the DL model predictions assigned higher probabilities of GON to those eyes that had thinner RNFL and lower SAP MD. This finding suggests that the model was able to identify early damage, although future longitudinal studies are needed to investigate the diagnostic accuracy of the DL model in eyes suspected of glaucoma.

This study has limitations. Our cohort consisted of a clinic-based population, meaning that validation for applications in population-based or opportunistic screening would be necessary. Also, the individuals in the glaucoma group were significantly older than the individuals in the normal group. Although we adjusted the analyses for age, such adjustment may not completely remove all the effects of the age difference between the two groups. Furthermore, the classification did not include risk factors, such as intraocular pressure, race, family history, and age. However, such information can be aggregated to estimate the pre-test probability and then one can apply the DL model to modify such probability. Finally, the DL algorithm would benefit from additional external validation on a population from a different geographical area.

In conclusion, we developed a DL algorithm to evaluate fundus photos for glaucomatous damage, based on a novel reference standard definition of GON that uses both structural and functional measures of disease. We demonstrated that the DL algorithm can produce large changes in the probability of disease in the majority of subjects, supporting its usefulness for decision-making. Furthermore, our proposed objective reference standard to define GON may increase the comparability of diagnostic studies across devices and populations, helping to improve the development and assessment of diagnostic tests in clinical practice.

## Methods

This was a retrospective study that used cross-sectional data from the Duke Glaucoma Registry, a database of electronic medical and research records at the Vision, Imaging, and Performance Laboratory at Duke University. The Duke Health Institutional Review Board approved this study, and a waiver of informed consent was granted due to the retrospective nature of this work. All methods adhered to the tenets of the Declaration of Helsinki for research involving human subjects and the study was conducted in accordance with regulations of the Health Insurance Portability and Accountability Act.

The visual field tests were performed using SAP with the 24-2 Swedish Interactive Threshold Algorithm (Carl Zeiss Meditec, Inc., Dublin, CA) protocol. Unreliable tests with more than 33% fixation losses or 15% false-positive errors were excluded. RNFL thickness measurements were obtained from peripapillary circle scans, acquired using the Spectralis SDOCT (Heidelberg Engineering, GmbH, Dossenheim, Germany). According to manufacturer recommendations, tests with a quality score lower than 15 were excluded. The fundus photos present in the database were acquired from two different cameras: Nidek 3DX (Nidek, Japan) and Visupac (Carl Zeiss Meditec, Inc., Dublin, CA). The image was retained if the optic disc was entirely visible in the photo, and if no artifacts were present.

### Objective definition of glaucoma

GON was defined based on the presence of corresponding structural and functional damage on SDOCT peripapillary RNFL scans and SAP, respectively, acquired within 6 months of each other. The definition considered the possibility of both global as well as localized losses. An eye was considered to have GON if any of the following were present:

1. Global RNFL thickness outside normal limits and abnormal SAP as defined by Glaucoma Hemifield Test (GHT) outside normal limits or Pattern Standard Deviation (PSD) with P < 5%,
2. At least one sector in the superior RNFL thickness (temporal-superior or nasal-superior) outside normal limits with a corresponding abnormality on SAP inferior hemifield, defined as inferior hemifield MD with P < 5%,
3. At least one sector in the inferior RNFL thickness (temporal-inferior or nasal-inferior) outside normal limits with a corresponding abnormality on SAP superior hemifield, defined as superior hemifield MD with P < 5%.

Superior and inferior SAP hemifield MD were calculated as the average of the total deviation values in each hemifield. The probability cut-offs for superior and inferior MD were derived from 462 eyes of 231 healthy subjects (72% white and 28% black), with mean age of 54.2 ± 15.0 (range: 21.1 to 88.2) years. None of these subjects were part of the cohort used to develop and test the deep learning model. Of note, as eyes with glaucoma may frequently have a dense arcuate defect occupying most of a hemifield, an approach using hemifield PSD to objectively characterize hemifield VF damage would have poor accuracy in diagnosis.

To be considered normal, or without GON, the SDOCT-SAP pair had to meet all of the following criteria:

1. Global RNFL thickness within normal limits,
2. RNFL thickness within normal limits for all sectors,
3. SAP GHT within normal limits,
4. SAP PSD probability “not significant”.

SDOCT-SAP pairs that did not meet criteria for GON or normal were considered suspects. These included eyes with only SDOCT abnormality or with only SAP abnormality. Although some suspects may in fact have early glaucoma, the lack of corresponding structural and functional damage leads to low specificity in the definition, which is undesirable in the context of developing an AI application for diagnosis and screening for glaucoma. Table 1 summarizes the criteria for the objective definition of GON.

### Subjective assessment

In order to assess the validity of the proposed objective definition of GON, two fellowship-trained glaucoma specialists evaluated a sample of pairs of SDOCT and SAP standard printouts and graded them as normal, suspect or glaucoma based on their clinical judgement. Disagreements between the two glaucoma specialists were resolved by the assessment of a third glaucoma specialist. The agreement between the subjective and objective classifications was investigated using weighted kappa statistics^10,11^.

### Deep learning model

A DL model was trained to predict the probability of GON on fundus photos, using as reference standard the proposed classification of GON defined based on SDOCT-SAP pairs. For each eye of each individual, all available fundus photos were matched with the closest SDOCT-SAP pair acquired within an interval of six months. Each SDOCT-SAP pair was classified as glaucoma, suspect or normal according to the objective definition of GON. Eyes that were suspected of glaucoma were not used for training the DL model.

Images were down sampled to 256 × 256 pixels and pixel values were rescaled to range from zero to one. Data augmentation was performed to decrease the effects of overfitting and increase generalizability and included: horizontal flips, random rotations (maximum of ten degrees), zoom (maximum factor of 1.1) and lighting (maximum factor of 20%) changes. No other pre-processing technique was performed.

The dataset was randomly divided at the subject level into 80% for training and validation of the model and 20% for final testing. Training of the DL algorithm was performed with transfer learning, using the Deep Residual Learning (ResNet50)^12^ architecture, pre-trained with the ImageNet dataset, with the head of the network adapted to our specific output task. The network was trained to minimize the cross-entropy loss function with minibatches of size 32, optimized with Adam algorithm^13^, for 300 epochs with early stopping based on the performance on validation sample. The best learning rate was found using the cyclical learning method^14^ with stochastic gradient descent with restarts. Gradient-weighted class activation maps^15^ were used to highlight the regions of the image that were most important for classification to visualize and understand the DL network predictions.

### Statistical analysis

The performance of the DL algorithm was assessed in overall test sample as well as according to disease severity using the Hodapp–Anderson–Parrish classification. ROC curves and the AUC were used to summarize the diagnostic accuracy. ROC analysis was adjusted for age^16–18^. Due to the presence of multiple tests for each eye and each subject, bootstrap resampling with resampling performed at the eye level was used for calculating CIs and statistical significance^19^.

Interval LRs were calculated to assess the impact of the results of the DL model in changing the probability of disease^6^. A LR is defined as the probability of a given test result in those with disease divided by the probability of that same test result in those without the disease^20,21^. The application of LRs in the interpretation of results of imaging instruments for glaucoma diagnosis has been detailed previously^22–24^. LRs represent the best way to incorporate diagnostic test results in clinical practice according to the principles of evidence-based medicine. The LR for a given test result indicates how much that result will change the probability of disease, going from a pre-test probability (i.e., probability of disease before the test) to a post-test probability. A value of one means that the test provides no additional information, and ratios above or below one increase or decrease the likelihood of disease, respectively. LRs greater than 10 or lower than 0.1 are associated with large effects on post-test probability, LRs from 5 to 10 or from 0.1 to 0.2 with moderate effects, LRs from 2 to 5 or from 0.2 to 0.5 with small effects, and LRs closer to one are insignificant^21^.

Development of the DL algorithm was performed in Python, and statistical analyses used Stata (version 15, StataCorp LP, College Station, TX). The alpha level (type I error) was set at 0.05.

## Supplementary Information

Supplementary Figure S1.

Supplementary Figure S2.

Supplementary Table S1.

Supplementary Table S2.

Supplementary Legends.
