# Supplementary figures and images for: An objective structural and functional reference standard in glaucoma
Source: Sci Rep. 2021 Jan 18;11:1752. doi: 10.1038/s41598-021-80993-3 (PMC7814070; doi:10.1038/s41598-021-80993-3)

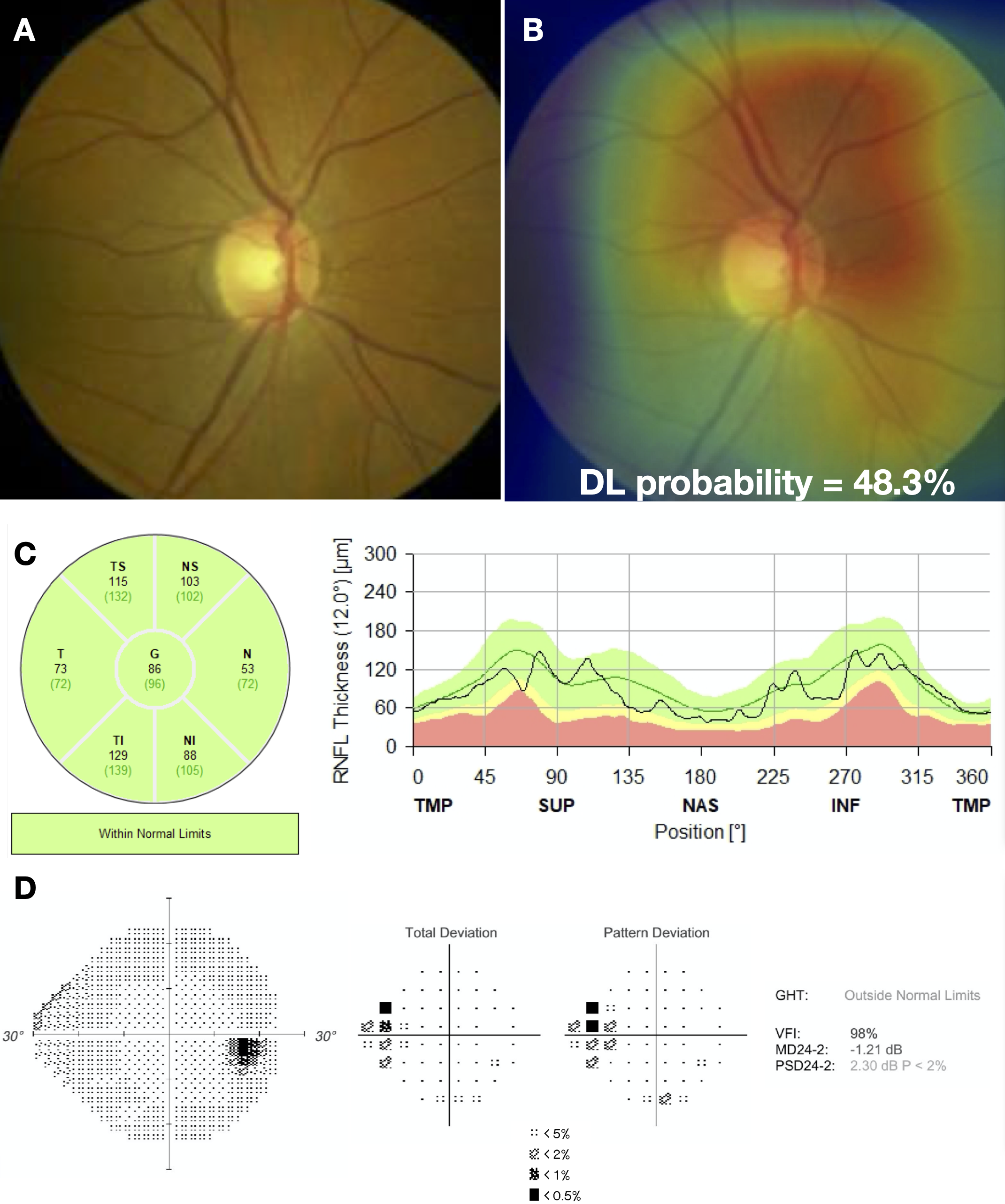

Supplement: Supplementary file 1 — Supplementary Figure S1. [file 41598_2021_80993_MOESM1_ESM.tiff]

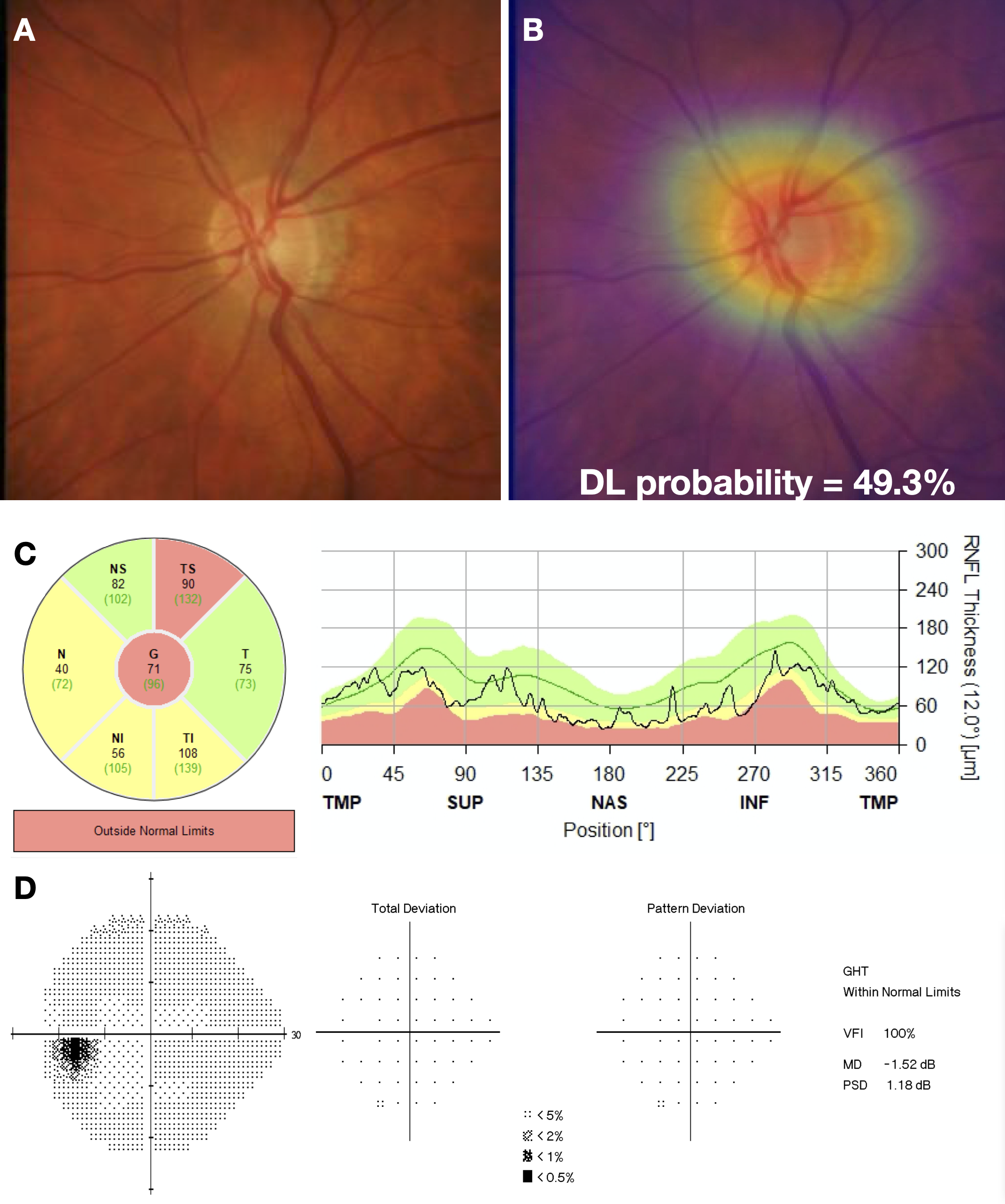

Supplement: Supplementary file 2 — Supplementary Figure S2. [file 41598_2021_80993_MOESM2_ESM.tiff]
