# Supplementary material for: An objective structural and functional reference standard in glaucoma
Source: Sci Rep. 2021 Jan 18;11:1752. doi: 10.1038/s41598-021-80993-3 (PMC7814070; doi:10.1038/s41598-021-80993-3)
Supplement: Supplementary file 3 — Supplementary Table S1. [file 41598_2021_80993_MOESM3_ESM.docx]

**An Objective Structural and Functional Reference Standard in Glaucoma**

**Eduardo B. Mariottoni, MD,^1,2^ Alessandro A. Jammal, MD,^1^ Samuel I. Berchuck, PhD,^1,3^ Leonardo S. Shigueoka, MD,^1^ Ivan M. Tavares, MD, PhD,^2^ Felipe A. Medeiros, MD, PhD^1,4*^**

1. Vision, Imaging and Performance (VIP) Laboratory, Duke Eye Center, Duke University, Durham, NC.
2. Department of Ophthalmology, Federal University of São Paulo, São Paulo, Brazil.
3. Department of Statistical Science and Forge, Duke University, Durham, North Carolina
4. Department of Electrical and Computer Engineering, Pratt School of Engineering, Duke University

Duke Eye Center, Department of Ophthalmology

Duke University

2351 Erwin Rd, Durham, NC 27701

Phone/Fax: +19196840201

E-mail: felipe.medeiros@duke.edu

**Supplementary Table S1.** Agreement between classifications based on the objective definition of glaucomatous optic neuropathy and the subjective assessment by glaucoma specialists.

|  | **Subjective assessment** | | |  |
| --- | --- | --- | --- | --- |
| **Objective classification** | **Normal** | **Suspect** | **Glaucoma** | **Total** |
| **Normal** | 250 | 33 | 0 | 283 |
| **Suspect** | 44 | 130 | 18 | 192 |
| **Glaucoma** | 0 | 54 | 196 | 250 |
| **Total** | 294 | 217 | 214 | 725 |
