# Supplementary material for: An objective structural and functional reference standard in glaucoma
Source: Sci Rep. 2021 Jan 18;11:1752. doi: 10.1038/s41598-021-80993-3 (PMC7814070; doi:10.1038/s41598-021-80993-3)
Supplement: Supplementary file 4 — Supplementary Table S2. [file 41598_2021_80993_MOESM4_ESM.docx]

**An Objective Structural and Functional Reference Standard in Glaucoma**

**Eduardo B. Mariottoni, MD,^1,2^ Alessandro A. Jammal, MD,^1^ Samuel I. Berchuck, PhD,^1,3^ Leonardo S. Shigueoka, MD,^1^ Ivan M. Tavares, MD, PhD,^2^ Felipe A. Medeiros, MD, PhD^1,4*^**

1. Vision, Imaging and Performance (VIP) Laboratory, Duke Eye Center, Duke University, Durham, NC.
2. Department of Ophthalmology, Federal University of São Paulo, São Paulo, Brazil.
3. Department of Statistical Science and Forge, Duke University, Durham, North Carolina
4. Department of Electrical and Computer Engineering, Pratt School of Engineering, Duke University

Duke Eye Center, Department of Ophthalmology

Duke University

2351 Erwin Rd, Durham, NC 27701

Phone/Fax: +19196840201

E-mail: felipe.medeiros@duke.edu

**Supplementary Table S2.** Diagnostic performance of the deep learning algorithm with objective classification and subjective assessment as reference standard.

|  | **Objective classification** | | **Subjective assessment** | |
| --- | --- | --- | --- | --- |
| **Disease severity** | **AUC (95% CI)** | **Sensitivity (at 95% specificity)** | **AUC (95% CI)** | **Sensitivity (at 95% specificity)** |
| **Overall** | 0.92 (0.88, 0.96) | 73.0% | 0.92 (0.88, 0.97) | 74.9% |
| **Early** | 0.89 (0.81, 0.96) | 63.6% | 0.84 (0.72, 0.96) | 59.8% |
| **Moderate** | 0.91 (0.85, 0.98) | 65.6% | 0.93 (0.85, 1.00) | 61.9% |
| **Severe** | 0.95 (0.91, 0.98) | 82.2% | 0.95 (0.91, 0.99) | 84.2% |

Abbreviations: AUC = area under the receiver operating characteristic curve; CI = confidence interval
