# Supplementary material for: An objective structural and functional reference standard in glaucoma
Source: Sci Rep. 2021 Jan 18;11:1752. doi: 10.1038/s41598-021-80993-3 (PMC7814070; doi:10.1038/s41598-021-80993-3)
Supplement: Supplementary file 5 — Supplementary Legends. [file 41598_2021_80993_MOESM5_ESM.docx]

**Supplementary Figure S1**. Example of a case classified as suspect. (A) illustrates the fundus photo used as input for the deep learning (DL) algorithm, which predicted a glaucoma probability of 48.3%. (B) shows the regions that were most important for the classification, in which the heatmap highlights diffusely the image, but mostly the superior half of the optic disc and the peripapillary region. Although the global and sectoral retinal nerve fibre layer thickness are within normal limits in the spectral domain optical coherence tomography (C), there is an apparent superior nasal step defect and the pattern standard deviation (PSD) is abnormal (P < 5%) in the standard automated perimetry (D).

**Supplementary Figure S2.** Example of a case classified as suspect. (A) illustrates the fundus photo used as input for the deep learning (DL) algorithm, which predicted a glaucoma probability of 49.3%. (B) shows the regions that were most important for the classification, in which the heatmap highlights diffusely the optic disc and a peripapillary atrophy. Although the spectral domain optical coherence tomography (C) is abnormal (global and temporal superior sectors outside normal limits, and 3 other sectors borderline), there is no defect in the standard automated perimetry (D).
